# Draft genome of three uropathogenic *Escherichia coli* strains

**DOI:** 10.1128/mra.00192-24

**Published:** 2024-06-06

**Authors:** Isabel Montserrat Cortez-de la Puente, Rosa del C. Rocha-Gracia, Patricia Lozano-Zaraín, Edwin Barrios-Villa, Margarita M. P. Arenas-Hernández

**Affiliations:** 1 Posgrado en Microbiología, Centro de Investigación en Ciencias Microbiológicas (CICM), Instituto de Ciencias (ICUAP), Benemérita Universidad Autónoma de Puebla (BUAP), Puebla, Pue, Mexico; 2 Laboratorio de Biología Molecular y Genómica, Departamento de Ciencias Químico-Biológicas y Agropecuarias, Universidad de Sonora, Campus Caborca, H. Caborca, Sonora, Mexico; University of Maryland School of Medicine, Baltimore, Maryland, USA

**Keywords:** genome sequence, genome analysis, uropathogenic *E. coli*, virulence factors, antibiotic resistance, evolution

## Abstract

Uropathogenic *Escherichia coli* (UPEC) remains the main etiological agent of urinary tract infections affecting females and males. The draft genome sequence of three strains of UPEC isolated from senior citizens and pregnant women in the state of Puebla, Mexico, is reported here.

## ANNOUNCEMENT

Urinary tract infections (UTI) are the most common bacterial infections, affecting over 150 million people worldwide each year (1, 2). Uropathogenic *Escherichia coli* (UPEC) is the main causative agent of UTI.

UPEC 123-I, 129-I, and 42GP, isolated from urine from patients with UTI and with risk factors, belong to a strain collection previously characterized. The strains possess classical UPEC virulence genes (Table 1) and were originally isolated by plating 10 µL of urinary sample on MacConkey agar and incubating aerobically overnight at 37°C. A lactose-positive colony was selected for identification by conventional biochemical test (3–5). Each strain, maintained at −70°C, was plated on MacConkey agar, and one lactose-positive colony was inoculated in 3 mL of LB broth and incubated overnight at 37°C. Genomic DNA was isolated using the Wizard Genomic DNA Purification Kit (Promega, USA). All protocols were conducted following the manufacturer’s instructions. The genomic DNA was resolved on 0.5% agarose gel to determine its integrity.

**TABLE 1:** Characteristics of three strains used in the present study and their virulence genes^*a*^

Strain	Sex	Patient age	Risk factor for UTI	Virulence genes	Reference
123-I	Female	75	Older age	*fimH, iucD, hlyA, sat, cnf-1, traT*	(3)
129-I	Male	73	Older age	*fimH, iha, fliC, iucD, hlyA, cnf-1, traT*	(4)
42GP	Woman	25	Pregnant	*fimH, iha, fliC, iucD, hlyA, sat, cnf-1, traT*	(5)

^*a*^ *fimH,* pili fimbriae adhesin type 1; *iha,* IrgA/siderophore homologous adhesin; *fliC,* flagellin; *iucD,* aerobactin; *hlyA,* α-hemolysin; *sat,* secreted autotransporter toxin; *cnf-1,* cytotoxic necrotizing factors; and *traT,* serum resistance.

Genomes were sequenced using both Illumina and Nanopore Technologies; in each case, distinct DNA extraction was used. Illumina DNA Prep Kit (Illumina, USA) was used to prepare the libraries, using IDT for Illumina Indexes with a target insert size of 460 bp. The NextSeq 500 platform was used for paired-end sequencing. The read length of the sequences was 2 × 149 paired end. Strains 123-I , 129-I, and 42GP have 8,936,357, 6,567,253, and 5,454,188 paired reads, respectively, with 530× (123-I and 42GP) and 321× (129-I) of depth.

The Native Barcoding 96 kit (SQK-NBD112.96) and Guppy base caller (Oxford Nanopore Technologies, USA) were used for sequencing with Oxford Nanopore Technology. The DNA was not fragmented or size selected for library construction; there was only a wash at the end of adapter ligation using the Large Fragment Buffer. The *N*_50_ for strain 123-I was 6,955 bp; for 129-I was 14,043 bp; and for 42GP was 4,220 bp. These strains have 783,911 (123-I), 143,194 (129-I), and 340,863 (42GP) reads. In the following analysis, default settings were used unless otherwise noted. For quality control and error correction of the raw reads, FastQC (v0.12.1) and trimmomatic (v0.36) were used (6, 7). *De novo* assembly of the reads was performed by SPAdes (v3.15.4) (8). The quality of assemblies was determined using QUAST (v4.6.0) (9). A total of 7 (123-I), 15 (129-I), and 12 (42GP) contigs were removed as they were below 200 bp in length. Strain 123-I has a genome size of 5,492,714 bp (19 contigs); 129-I has 5,466,769 bp (41 contigs); and 42GP has 5,333,327 bp (18 contigs). The genomes have an *N*_50_ of 2,445,443 (123-I), 3,209,856 (129-I), and 3,707,725 bp (42GP). The strain 123-I has 50.71% of GC, while strains 129-I and 42GP have 50.69% of GC. The annotation was carried out following the Prokaryotic Genome Annotation Pipeline v6.6 of the NCBI (10).

## Data Availability

The draft genomes of UPEC 123-I, 129-I, and 42GP were deposited in the National Center for Biotechnology Information (NCBI) under the accession numbers JBAISI000000000, JAZGOG000000000, and JAZGOH000000000, respectively. In addition, all raw reads were submitted to NCBI’s SRA under the accession numbers SRR27907043 and SRR27889912 for strain 123-I; SRR27899061 and SRR27899060 for strain 129-I; and SRR27899059 and SRR27899058 for strain 42GP.
